# Synthesis and Characterization of Methoxy-Exfoliated Montmorillonite Nanosheets as Potential Carriers of 5-Fluorouracil Drug with Enhanced Loading, Release, and Cytotoxicity Properties

**DOI:** 10.3390/molecules28155895

**Published:** 2023-08-05

**Authors:** Mashael D. Alqahtani, May N. Bin Jumah, Abdulrahman Al-Hashimi, Ahmed A. Allam, Mostafa R. Abukhadra, Stefano Bellucci

**Affiliations:** 1 Department of Biology, College of Science, Princess Nourah bint Abdulrahman University, P.O. Box 84428, Riyadh 11671, Saudi Arabia; 2 Botany and Microbiology Department, College of Science, King Saud University, Riyadh 11451, Saudi Arabia; 3 Zoology Department, Faculty of Science, Beni-Suef University, Beni-Suef 62514, Egypt; 4 Geology Department, Faculty of Science, Beni-Suef University, Beni-Suef 65211, Egypt; 5 Materials Technologies and Their Applications Laboratory, Geology Department, Faculty of Science, Beni-Suef University, Beni-Suef 62514, Egypt; 6 INFN-Laboratori Nazionali di Frascati, Via E. Fermi 54, 00044 Frascati, Italy

**Keywords:** bentonite, exfoliation, methanol, 5-fluorouracil, loading, cytotoxicity

## Abstract

Natural bentonite clay (BE) underwent modification steps that involved the exfoliation of its layers into separated nanosheets (EXBE) and further functionalization of these sheets with methanol, forming methoxy-exfoliated bentonite (Mth/EXBE). The synthetically modified products were investigated as enhanced carriers of 5-fluorouracil as compared to raw bentonite. The modification process strongly induced loading properties that increased to 214.4 mg/g (EXBE) and 282.6 mg/g (Mth/EXBE) instead of 124.9 mg/g for bentonite. The loading behaviors were illustrated based on the kinetic (pseudo-first-order model), classic isotherm (Langmuir model), and advanced isotherm modeling (monolayer model of one energy). The Mth/EBE carrier displays significantly higher loading site density (95.9 mg/g) as compared to EXBE (66.2 mg/g) and BE (44.9 mg/g). The loading numbers of 5-Fu in each site of BE, EXBE, and Mth/EXBE (>1) reflect the vertical orientation of these loaded ions involving multi-molecular processes. The loading processes that occurred appeared to be controlled by complex physical and weak chemical mechanisms, considering both Gaussian energy (<8 KJ/mol) as well as loading energy (<40 KJ/mol). The releasing patterns of EXBE and Mth/EXBE exhibit prolonged and continuous properties up to 100 h, with Mth/EXBE displaying much faster behaviors. Based on the release kinetic modeling, the release reactions exhibit non-Fickian transport release properties, validating cooperative diffusion and erosion release mechanisms. The cytotoxicity of 5-Fu is also significantly enhanced by these carriers: 5-Fu/BE (8.6% cell viability), 5-Fu/EXBE (2.21% cell viability), and 5-Fu/Mth/EXBE (0.73% cell viability).

## 1. Introduction

In the next few years, it is expected that non-contagious diseases, mostly cancer, will account for 75% of all reported mortality worldwide [1,2]. Colorectal cancer is a common invasive type of cancer that affects about 13% of cancer patients worldwide. It represents one of the two foremost causes of death, which raises the global death rate [3,4,5]. The initial stage of colorectal cancer is a polyp that develops within the mucosal layers and then spreads to the current submucosa and surrounding tissues. In the later stages of colorectal cancer, the malignant cells that are produced migrate to the nearby tissues and lymph nodes [6]. As a result, the establishment of effective and safe medicines capable of suppressing the growth of tumor cells without noticeable or significant adverse responses constitutes an urgent problem and an active topic of study in the pharmaceutical and academic communities [5,7].

Chemotherapies of different types are commonly utilized for inhibiting the growth and proliferation of cancer cells [8,9]. The frequently administered chemotherapies effectively impede DNA replication and generate a significant level of oxidative stress, which damages malignant cells [5,10]. Unfortunately, the vast majority of presently utilized chemotherapies possess toxic effects on healthy cells and have several serious side effects, especially at high doses, such as nausea, renal failure, and bone marrow depletion. As a result, numerous studies have been performed to improve the safety, biological compatibility, curative characteristics, and selectivity of the different types of traditional chemotherapies [11]. It was hypothesized that this improvement may be achieved by either developing innovative forms of anticancer medications or improving the efficacy and biosafety of already existing traditional forms [3].

One of the most frequently used chemotherapy medications is 5-fluorouracil (5-Fu), which is effective in treating a variety of tumor cell types, including rectum, breast, colorectal, and stomach malignancies [1,12]. Unfortunately, similar to the majority of chemotherapies, the use of 5-Fu has a number of negative aspects related to its poor specificity, strong diffusion rate, and weak solubility, in conjunction with the highly toxicological effects of its overdoses [13,14]. 5-Fu is extremely toxic to the nervous, digestive, cardiovascular, blood, and skin systems [12,15]. As a consequence, numerous new delivery systems have been investigated as effective strategies to increase the curative activity, medicinal value, solubility, release rate, and specificity of 5-Fu [1,11,16]. It has been highly recommended to manage the delivered doses at predetermined intervals and at regulated rates to prevent the frequently reported health obstacles and prolong the interaction period [17,18,19]. Additionally, this may have a considerable positive impact on the compliance of patients and curative characteristics besides its reduction impact on the drug degradation rate, keeping the concentration at the required level [18,19]. As possible delivery systems for traditional chemotherapies, zeolite, mesoporous silica, polymers, CaO, layered double hydroxide (LDH), and montmorillonite have all been investigated successfully [4,5,11,20,21]. The prior materials significantly improved the permeability and retention properties of the medication [4,5,9,17]. Clay-based materials such as kaolinite, sepiolite, montmorillonite, halloysite, and vermiculite have been extensively studied as suitable carriers for 5-Fu. The majority of clay minerals have distinct layered aluminosilicate frameworks and are characterized by large natural reserves, high ion exchange properties, excellent biocompatibility, substantial adsorption capacity, low toxicology, excellent chemical stability, low cost, and flexible chemical structure [12,22,23].

Bentonite and its modified varieties have been widely investigated as very effective adsorbents and drug carriers [22,24]. Bentonite is a commonly used geological term to describe a form of widely distributed sedimentary rock made mostly of smectite clay minerals, particularly montmorillonite, with additional types of clay minerals as well as non-clay minerals as mineralogical impurities [23,25]. A number of studies have been carried out to improve the surface characteristics and physicochemical qualities of raw bentonite, particularly as a potential adsorbent of organic molecules and a carrier of drugs [22,26,27]. The modification or hybridization of bentonite involved thermal activation, alkaline activation, acid leaching, thermal treatment, metal pillaring, metal oxide integration, exfoliation, polymer intercalation, and organic hybridization (CTAB, starch) [22,26,27,28].

Methoxy-modified clay-based structures have recently been identified as more sophisticated types of organophilic clay that have a strong affinity for soluble organic molecules and improved adsorption characteristics either as an adsorbent or as a drug carrier [12,29]. By environmental grafting processes of the interior surface hydroxyl groups under room conditions, the clay layers can intercalate with the alcohol molecules [12,30,31]. Despite the fact that a number of investigations have been conducted to explore the physicochemical characteristics of methoxy-kaolinite, no prior investigations have been accomplished to assess the characteristics of methoxy-bentonite, especially as a drug carrier [29]. The preparation of methoxy-kaolinite requires a stage of pretreatment to overcome the impact of the existing hydrogen bonds between the structural units of the kaolinite, which entails extra chemicals and a variety of experimental processes [13,29]. Compared to kaolinite, montmorillonite has highly interchangeable hydroxyl layers that allow the integration of organic molecules between its layered units, which allows the incorporation of the methanol molecules freely without additional prior modification procedures [21,29]. It was thus predicted that modifying bentonite-layered units with methanol would provide a simple, multifunctional, and organophilic hybrid structure with improved physicochemical qualities.

Furthermore, in the past few years, the exfoliation and separation of clay-layered units into individual and single layers have been mentioned as an effective modification approach that produces new clay nanostructures with enhanced surface reactivity, surface area, and adsorption capacity [5,21]. As a drug carrier and anticancer agent, exfoliated bentonite (EXBE) functionalized as individual layers with methanol molecules to produce methoxy-exfoliated bentonite (Mth/EXBE) was expected to yield a novel structure with improved surface area, loading properties, surface reactivity, release profiles, and cytotoxic characteristics. Therefore, the presented work includes the production of methoxy-exfoliated bentonite (Mth/EXBE) as an innovative nano-delivery system for 5-fluorouracil medication that has improved loading, release, and cytotoxic characteristics. Theoretical kinetic and equilibrium studies were used to evaluate the loading and release mechanisms, in addition to the cytotoxicity properties of the studied materials as anticancer agents against colorectal cancer cells (HCT-116).

## 2. Experimental Work

### 2.1. Materials

The bentonite precursor samples (54.82% (SiO_2_), 2.5% (MgO), 9.5% (Fe_2_O_3_), 17.56% (Al_2_O_3_), 2.4% (CaO), 2.6% (Na_2_O), 1.45% (TiO_2_), and 9.2% (LOI)) were delivered from a natural bentonite quarry, Western Desert, Egypt. The chemicals applied during the different synthesis procedures were NaF, (NaPO_3_)_6_, dimethyl sulfoxide (DMSO), and methanol. All these chemicals were of analytical grade and were purchased from Sigma-Aldrich, Cairo, Egypt, to be used during the exfoliation procedures and the methanol functionalization steps. 5-fluorouracil drug (analytical grade >99%) was obtained from Sigma-Aldrich Company, Egypt, to be used during the investigated loading, release, and cytotoxicity experiments.

### 2.2. Synthesis of Methoxy-Exfoliated Bentonite (Mth/EXBE)

The liquid-phase stripping approach was used to complete the conventional bentonite exfoliation processes. First of all, 10 g of the ground bentonite was mixed homogeneously for 90 min in a NaF aqueous solution (NaF (0.4 g) + 200 mL) at an ambient temperature of about 80 °C while being stirred at 500 rpm. The previously produced slurry was then supplemented with (NaPO_3_)_6_ (0.05 g) and afterward treated with ultrasound waves for 120 min at a power setting of 240 W. Following the sonication procedure, the formed supernatant was kept at room temperature for 24 h before being centrifuged at a rapid rate for 30 min (5000 rpm) to separate the bentonite colloidal particles, which are the exfoliated product (EXBE). Following this, the washed EXBE was blended with 100 mL of methanol over 48 h in a glass autoclave at room temperature. The homogenization between EXBE and methanol was performed by employing a magnetic stirring device at 500 rpm and an ultrasound source at 240 W. The product was then thoroughly removed and washed once more. It was then gently dried for 12 h at 50 °C and marked as Mth/MXBE to be incorporated into further characterization and experimentation.

### 2.3. Analytical Techniques

Based on the obtained XRD patterns, the crystallinity and the main crystal phases were determined using a PANalytical-Empyrean X-ray diffractometer across a detection range of 0 to 70°. A Fourier transform infrared spectrometer (FTIR8400S; Shimadzu, Tokyo, Japan) was used to discriminate between the chemical compositions of BE, EXBE, and Mth/EXBE within the determination frequency range of 400 cm^−1^ to 4000 cm^−1^. SEM images were captured using a scanning electron microscope (Gemini, Zeiss Ultra 55) immediately after coating the BE, EXBE, and Mth/BE particles with thin gold films. The HRTEM images, which were produced using a transmission electron microscope (JEOL-JEM2100) at an accelerating voltage of 200 kV, were used to study the internal structure of BE, EXBE, and Mth/EXBE. A Beckman Coulter SA3100 surface area analyzer and associated N_2_ adsorption/desorption isotherms were used to measure the surface area and porosity of BE, EXBE, and Mth/EXBE.

### 2.4. 5-Fu Loading Studies

The loading of 5-Fu into BE, EXBE, and Mth/EXBE was evaluated based on the basic parameters that regulate the 5-Fu loading dosage as well as the maximum loading capacities. The key variables that were assessed throughout the investigation were pH (3–9), loading duration (1–24 h), and 5-Fu starting concentrations (100–800 mg/L). The BE, EXBE, and Mth/EXBE nanoparticles were successfully homogenized within the evaluated 5-Fu aqueous solutions (100 mL) using a vortex rotator apparatus (VM-300; Gemmy Industrial Corp., Taipei, Taiwan). The BE, EXBE, and Mth/EXBE particulates were separated from the tested 5-Fu solutions by filtering these particles through Whatman paper after each test’s equilibration period. The concentration of the residual 5-Fu was subsequently assessed using a UV-Vis spectrophotometer at an adjustable wavelength (ʎ _(max)_ = 270 nm). Based on Equation (1), the rest of the 5-Fu levels were used to calculate the loading capabilities of BE, EXBE, and Mth/EXBE in mg/g. The studies of 5-Fu loading into BE, EXBE, and Mth/EXBE were performed in triplicate, and the estimated median values with standard deviations of 4.2% are presented in the study data.

(1) $$\mathrm{Loaded}\;\mathrm{drug}\;\left(\mathrm{mg}/g\right)=\frac{\left(\mathrm{Initial}\;\mathrm{concentration}-\mathrm{Residual}\;\mathrm{concentration}\right)\times \;\mathrm{solvent}\;\mathrm{volume}}{\mathrm{Carrier}\;\mathrm{weight}}$$

### 2.5. The Release Studies

Two distinct biochemical buffers (gastric fluid, pH 1.2, and intestinal fluid, pH 7.4) at 37.5 °C were used to evaluate the 5-Fu-releasing behaviors of BE, EXBE, and Mth/EXBE. Five hundred mL of the tested release buffers was thoroughly mixed with the 5-Fu-loaded BE, EXBE, and Mth/EXBE particulates (100 mg/g). The loaded samples were prepared according to the estimated best loading conditions (time: 24 h; dose: 20 mg; temperature: 20 °C; pH: 9; volume: 100 mL; concentration 100 mg/L). The two different buffers and the 5-Fu-loaded BE, EXBE, and Mth/EXBE particulates were homogenized separately using a DISTEK dissolution apparatus (Model-2500, DISTEK Co.; North Brunswick Township, NJ, USA) for 120 h at 200 rpm, which was the adjustable vessel’s rotating speed. Using a UV-Vis spectrophotometer, samples of the two distinct buffered solutions (5 mL) were analyzed to track the percentages of 5-Fu diffused from BE, EXBE, and Mth/EXBE. These samples were periodically obtained from the total volumes of the releasing buffer solutions. To maintain the volumes at precisely the same values during the whole release duration, the bulk releasing buffers were instantaneously refilled with the frequently collected samples. The 5-Fu release tests were carried out in triplicate, and the average values with a standard deviation of less than 3.6% computed using Equation (2) are presented in the study.

(2) $$\mathrm{Drug}\;\mathrm{release}\;\left(\%\right)=\frac{\mathrm{The}\;\mathrm{amount}\;\mathrm{of}\;\mathrm{Released}\;5\text{–}\mathrm{Fu}\;}{\mathrm{Amount}\;\mathrm{of}\;\mathrm{loaded}\;5\text{–}\mathrm{Fu}}\times 100$$

### 2.6. In Vitro Cytotoxicity

#### 2.6.1. Cell Line

The colorectal cancer cell line (HCT-116) was obtained from the American Type Culture Collection (ATCC, Rockville, MD, USA) and assessed as the target cancer cells during the conducted cytotoxic assays. Gentamycin, HEPES buffer, 0.25% trypsin-EDTA, dimethyl sulfoxide (DMSO), fetal bovine serum, DMEM, RPMI-1640, and 3(4, 5-dimethylthiazol-2-yl)-2.5 diphenyltetrazolium bromide (MTT 99%) were the key chemical reagents used in the incubation process and cytotoxicity testing.

#### 2.6.2. In Vitro Cytotoxicity

The cancerous HCT-116 cells were first cultured at 37 °C and 5% CO_2_ within RPMI-1640 media containing 50 µg/mL gentamycin and 10% fetal calf serum. The tumor cells (5 × 10^4^ cells/well) were incubated for three weeks prior to being submerged in Corning^®^ 96-well plates for 24 h. The cells were subsequently treated with specific doses of the 5-Fu-loaded EXBE and Mth/EXBE (50 µg/L, 100 µg/L, 200 µg/L, 300 µg/L, 400 µg/L, and 500 µg/L) as suspensions in 50 µL, after which they were cultivated for an additional 24 h. During the incubation period, the total number of viable cells produced was counted using the common MTT cell proliferation test. After the completion of the incubation period, the included culture medium was successfully removed and a fresh medium (100 µL of RPMI) was added in its stead. The newly added medium was well mixed with the MTT (10 µL; 12 mM), and the mixture was cultivated for another 5 h to examine if formazan with a distinct purple color had developed. After that, 50 L of DMSO solution was used to effectively dissolve the formed formazan. The optical density (OD) of the cells that were cultured throughout the process was measured in the last step using a microplate that was adjusted to a certain wavelength of 590 nm. The calculated values were used to calculate the cell viability % using Equation (3) [11].

(3) $$\mathrm{Cell}\;\mathrm{viability}\;\left(\%\right)=\frac{\mathrm{Mean}\;\mathrm{OD}}{\mathrm{Control}\;\mathrm{OD}}\times 100$$

## 3. Results and Discussion

### 3.1. Characterization of the Carriers

**Figure 1:**
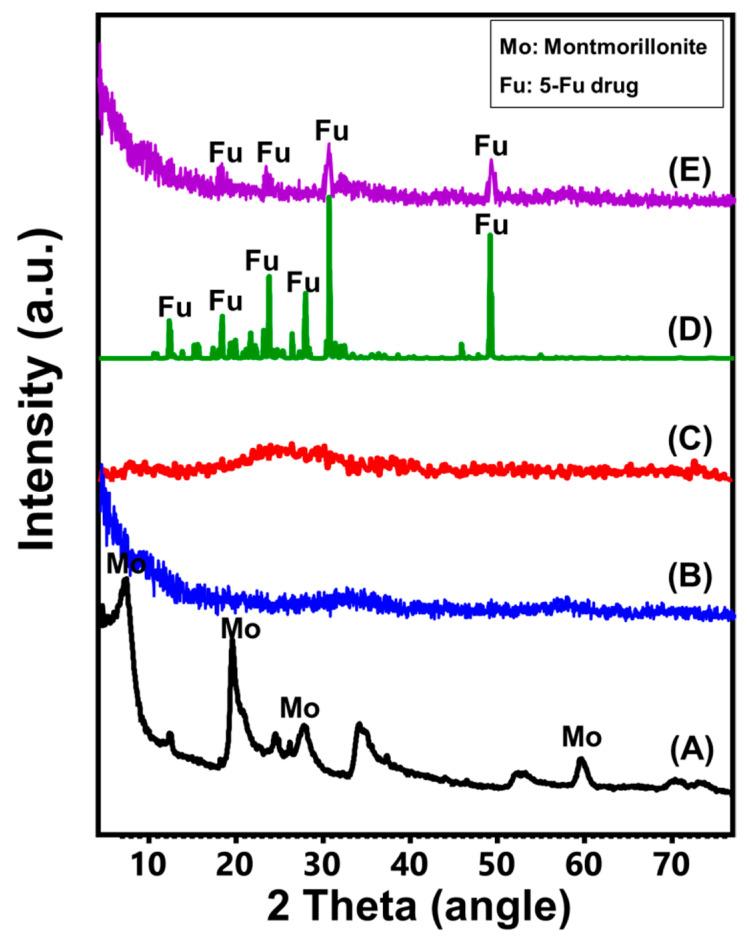
XRD patterns of bentonite (BE) (**A**), exfoliated bentonite (EXBE) (**B**), methoxy-exfoliated bentonite (Mth/EXBE) (**C**), 5-Fu drug (**D**), and 5-Fu-loaded Mth/EXBE particles (**E**).

The examined XRD pattern of the utilized bentonite material demonstrated that the sample was enriched in montmorillonite as the predominant clay phase, exhibiting its characteristic peaks around 6.55°, 19.85°, 25.10°, and 28.35° (cards No. 00-058-201 and No. 00-003-0010) (Figure 1A). After the exfoliation procedures, the bentonite sample’s pattern was noticed as a wide peak with no convincing evidence of the existence of crystalline materials (Figure 1B). The separation of the montmorillonite layers and disintegration of the lattice framework indicated successful exfoliation and delamination of the montmorillonite layered units. The chemical modification of the exfoliated bentonite layers by the methanol molecules shows no discernible structural impact, and the EXBE product retains its non-crystalline characteristics (Figure 1C). The recognized XRD pattern of 5-Fu-loaded Mth/EXBE (Figure 1D) was compared with the obtained pattern of free 5-Fu powder (Figure 1C). The recognized XRD pattern of the loaded sample demonstrates an extensive reduction in the intensities of the diffraction peaks of 5-Fu and a considerable deviation in the positions and an increase in their broadness. This suggests the notable impact of the used carrier in promoting the amorphization of the drug crystals and in turn their solubility properties (Figure 1D).

**Figure 2:**
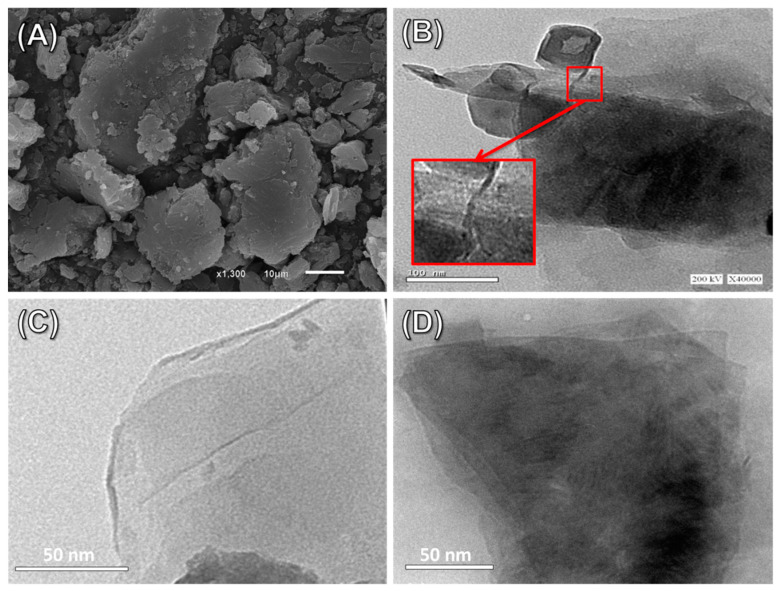
SEM image of BE particles (**A**), HRTEM image of BE particles (**B**), HRTEM image of synthetic EXBE particles (**C**), and HRTEM image of synthetic Mth/EXBE particles (**D**).

The utilized bentonite particulates were observed in the SEM images as clumped sheets packed on top of each other, forming aggregates and agglomerated massive particles (Figure 2A). The observable small chunks on the surfaces of the bentonite layers might be assigned to the present impurities rather than the clay minerals (Figure 2A). The layered structural units of montmorillonite, the essential component of bentonite, were readily discernible in the HRTEM image, which showed the mineral’s distinctive multilayered form, which is known as the lattice finger structure (Figure 2B). After the exfoliation procedures, the bentonite’s HRTEM images demonstrated the effective separation of the compressed montmorillonite layered units into independent and individual aluminosilicate layers that had a greater positive influence on the surface reactivity and area (Figure 2C). The observed HRTEM images of fabricated methoxy-exfoliated bentonite (Mth/EXBE) still show the stripping of the bentonite compacted layers into individual and separated layers, in addition to the notable observation of irregular portions of dark gray color that may be attributed to the organic methanol molecules and their interaction with the silicate structure of the bentonite (Figure 2D). The exfoliation and functionalization processes are associated with a significant increment in the surface area from 91 m^2^/g for bentonite to 141.4 m^2^/g and 143.2 m^2^/g for EXBE and Mth/EXBE, respectively.

**Figure 3:**
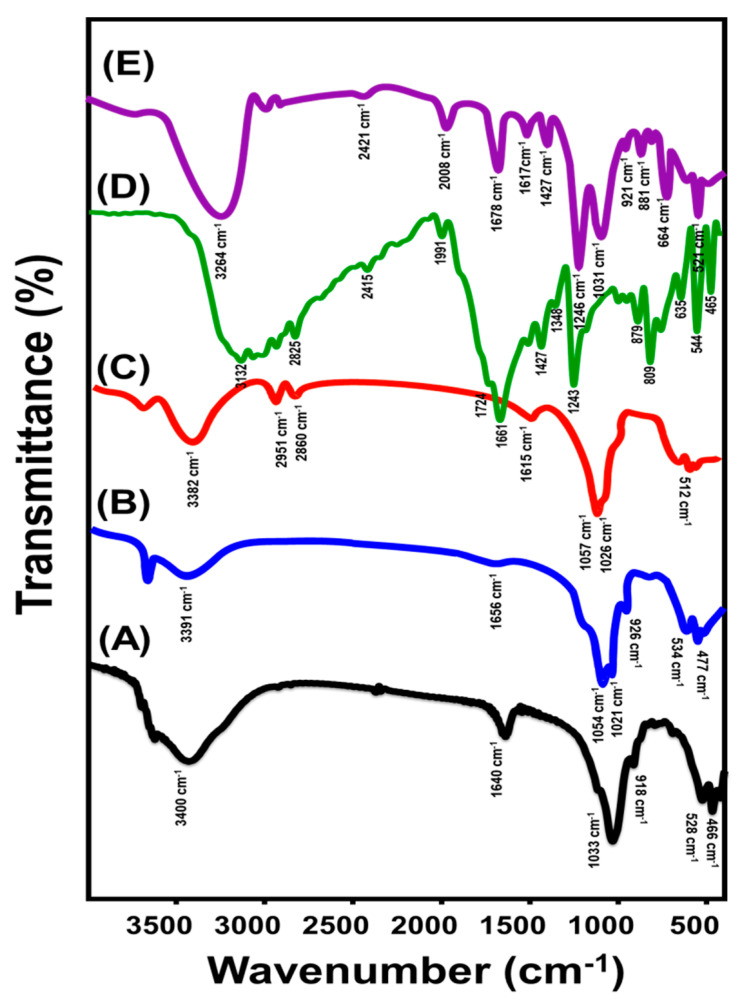
FT–IR spectra of bentonite (BE) (**A**), exfoliated bentonite (EXBE) (**B**), methoxy-exfoliated bentonite (Mth/EXBE) (**C**), 5-Fu drug (**D**), and 5-Fu-loaded Mth/EXBE particles (**E**).

Based on the FT-IR spectra, the natural bentonite exhibited predominant groups of crystalline OH, interstitial molecules of water, Si-O, and Al-O, which correspond to the absorption bands at 3400 cm^−1^, 1640 cm^−1^, 1000 cm^−1^, and 918 cm^−1^, respectively [21,23]. Additionally, the observed weak bands in the range from approximately 400 cm^−1^ to 1000 cm^−1^ identify the distinctive bands of Si-O-Al, Si-O-Mg, and Mg-Fe-OH [21,23] (Figure 3A). The detected spectrum of EXBE shows absorption bands that are comparable to those observed in raw bentonite, but with significant variations in their marked positions, a decline in their intensities, and an absence of smaller bands (Figure 3B). This reflects the predicted deformation of the bentonite’s structural octahedron and tetrahedron units and the effective exfoliation of those units into individual or single sheets [32,33] (Figure 3B). After the modification of EXBE with methanol, the detected FT-IR spectrum indicated a decrease in the intensities of the marked bands of the interior surficial hydroxyl groups. This illustrates the integration of these chemical groups (Al-OH) during the modification of bentonite layered units with methanol (Figure 3C) [12,29]. Furthermore, the marked bands at 2951 cm^−1^ and 2860 cm^−1^ correspond to the grafted methanol groups (C-H stretching) (Figure 3C) [12,30]. The above, in addition to the observable shifting of the corresponding bands of the aluminosilicate functional groups of bentonite layers, signifies the successful interaction between the EXBE units and the methanol molecules and the formation of methoxy-exfoliated bentonite (Mth/EXBE). The FT-IR spectrum of 5-Fu-loaded Mth/EXBE (Figure 3E) was compared with the spectrum of the free 5-Fu drug (Figure 3D). The spectrum demonstrated fluctuation in the reported bands of Mth/EXBE in addition to the existence of some bands related to the chemical structure of the 5-Fu drug such as C-F stretching (1240 cm^−1^), C-N stretching (1427 cm^−1^), and C=O bending (1678 cm^−1^), confirming the successful loading of drug molecules (Figure 3E)

### 3.2. Loading of 5-Fu Drug

#### 3.2.1. Influence of the Encapsulation Parameters

##### Effect of pH

**Figure 4:**
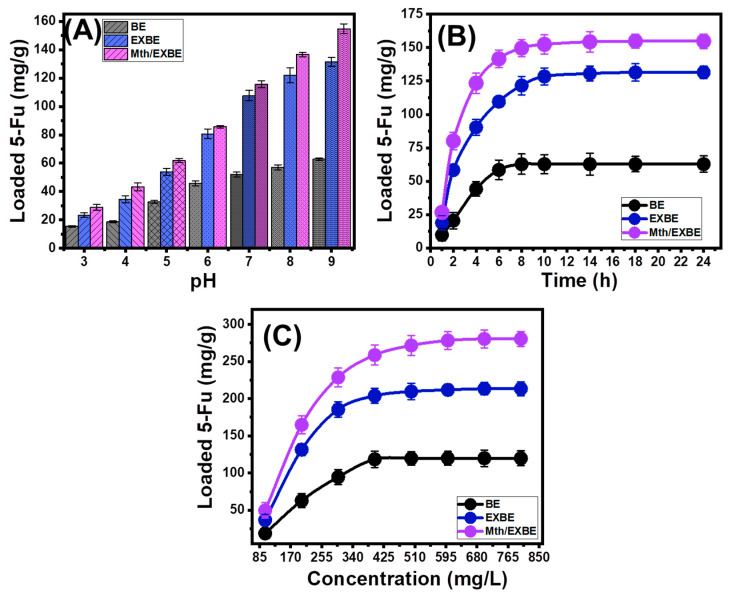
Effect of the main loading variables on the loading capacities of BE, EXBE, and Mth/EXBE including the effect of pH (**A**), loading duration (**B**), and tested 5-Fu concentration (**C**).

The experimental impact of the solution’s pH on the 5-Fu loading properties of BE, EXBE, and Mth/EXBE has been investigated from pH 3 to pH 9 at adjustable levels for the other parameters that may influence the behaviors (dose: 20 mg; 5-Fu concentration: 200 mg/L; temperature: 20 °C; volume: 100 mL; time: 24 h). It has been demonstrated that high pH conditions greatly enhance the 5-Fu’s ability to be encapsulated in BE, EXBE, and Mth/EXBE (Figure 4A). This can be described from pH 3 (BE (15.4 mg/g), EXBE (23.3 mg/g), and Mth/EXBE (28.9 mg/g)) to pH 9 (BE (62.8 mg/g), EXBE (131.5 mg/g), and Mth/EXBE (154.8 mg/g)) (Figure 4A). Therefore, it was suggested to employ the processes of 5-Fu loading into BE, EXBE, and Mth/EXBE at basic pH levels. The ionization characteristics of 5-Fu as well as the predominant surficial charges of BE, EXBE, and Mth/EXBE are frequently influenced by the solutions’ adjusted pH. The molecular composition of the 5-Fu medicine has significant ionization aspects under higher pH settings (alkaline) as compared to its properties under acidic to neutral environments [11,22]. The increase in ionization rates increases the 5-Fu loading affinities under alkaline conditions by enhancing the mobility properties, diffusion behaviors, and interaction chances between the drug ions and the distributed active loading sites of BE, EXBE, and Mth/EXBE.

##### Loading Duration

Experimentally, the influence of the loading interval on the BE, EXBE, and Mth/EXBE performances was investigated throughout a time range of 1 to 24 h, keeping the other influencing factors at constant values (pH: 9, 5-Fu concentration: 200 mg/L, temperature: 20 °C, dosage: 20 mg, volume: 100 mL). The 5-Fu loading efficiencies of BE, EXBE, and Mth/EXBE exhibit substantial improvements in terms of the loading rates as well as the 5-Fu loading amounts in mg/g with a steady increase in the evaluated duration (Figure 4B). This enhancement effect can be observed from 1 h to 8 h for BE and from 1 h to 10 h for EXBE and Mth/EXBE; after that, a further extension of the test’s period has no significance for the loading rate or the quantity of loaded 5-Fu, and the plots display equilibrium states with nearly the same values (Figure 4B). These properties reflect the equilibrium conditions of BE, EXBE, and Mth/EXBE and correspond to their equilibrium loading capacities (BE (62.8 mg/g), EXBE (131.5 mg/g), and Mth/EXBE (154.8 mg/g)) (Figure 4B). The expected availability of the active sites in the free states and in extensive numbers on the BE, EXBE, and Mth/EXBE surfaces in the early stages resulted in a substantial increase in the loading efficiencies and a quick rise in the loaded amounts of 5-Fu [13]. As the duration of the experiments increases, more and more 5-Fu is loaded successfully into the free sites of BE, EXBE, and Mth/EXBE. This in turn leads to the saturation and occupation of these sites and dramatically decreases their accessibility. Thus, the experimentally determined 5-Fu loading rates clearly dropped after a certain period of time, and the 5-Fu loading characteristics of BE, EXBE, and Mth/EXBE show little to no improvement. The equilibrium states of BE, EXBE, and Mth/EXBE were established once the 5-Fu molecules filled all the accessible loading sites [34].

##### 5-Fu Concentration

The experimental effects of the various concentrations of 5-Fu on the loading qualities of BE, EXBE, and Mth/EXBE were studied throughout a range of 100 to 800 mg/L while maintaining the other parameters at constant values (time: 24 h; dose: 20 mg; temperature: 20 °C; pH: 9; volume: 100 mL). The maximum capacities of BE, EXBE, and Mth/EXBE to load 5-Fu, as well as their equilibrium behaviors, are mostly determined by their initially tested 5-Fu concentrations. The overall quantities of 5-Fu ions that were loaded into BE, EXBE, and Mth/EXBE increased notably in the context of the high concentrations of the initially tested 5-Fu ions (Figure 4C). The driving forces and diffusion characteristics of the 5-Fu ions were considerably enhanced when they were present in a highly concentrated state at a certain volume. This encourages contacts and chemical interactions that occur between the 5-Fu ions and the dominant binding sites of BE, EXBE, and Mth/EXBE [35,36]. This, in turn, improves the 5-Fu loading performance of BE, EXBE, and Mth/EXBE up to certain concentrations (400 mg/L (BE), 500 mg/L (EXBE), and 600 mg/L (Mth/EXBE)) (Figure 4C). After these levels, any increase in the estimated 5-Fu concentration has no effect on the quantifiable loads of 5-Fu, which typically denote the equilibrium loading states of BE, EXBE, and Mth/EXBE (Figure 4C). The maximum capacities of BE, EXBE, and Mth/EXBE for loading 5-Fu were identified at these states (119.8 mg/g for BE, 213.3 mg/g for EXBE, and 280.5 mg/g for Mth/EXBE) (Figure 4C). The substantially enhanced 5-Fu loading properties of EXBE and Mth/EXBE in contrast with BE were attributed to a variety of parameters, such as (1) the documented rise in surface area following the modification process, (2) the organophilic characteristic of Mth/EXBE in contrast to the hydrophilicity of BE, which improves its surface affinity to the 5-Fu organic molecules, and (3) an appreciable rise in the numbers of the functioning loading sites after the alteration steps.

#### 3.2.2. Loading Mechanism

##### Kinetic Properties

Intra-Particle Diffusion Behavior

**Figure 5:**
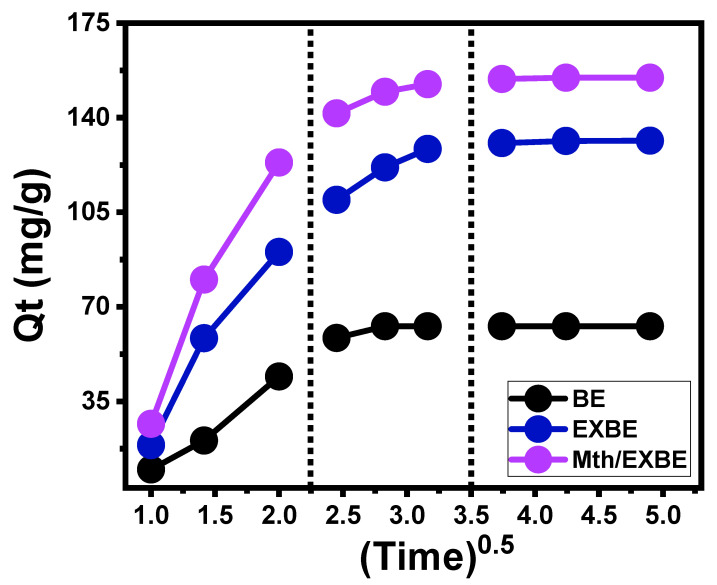
The intra-particle diffusion curves of the occurred 5-Fu loading processes for BE, EXBE, and Mth/EXBE (vertical dashes refer to the different segments of the curves).

The processes that load 5-Fu into BE, EXBE, and Mth/EXBE exhibit intra-particle diffusion curves with segment-like properties, comprising three discrete phases with no crossovers with the beginning points of these curves (Figure 5). This demonstrates the 5-Fu loading through collaborative mechanisms as well as the significant influence of drug ion diffusion in the direction of the reactive surfaces of BE, EXBE, and Mth/EXBE [36,37]. This may comprise (A) loading processes performed by the surficial or exterior active sites (border), (B) mechanistic effects of intra-particle diffusion processes, and (C) the effect of the attended equilibration or saturation states [38]. The existence of the first step in the curves implies that exterior loading mechanisms were active and dominant as the investigation began, and the total number of the existing exterior sites strongly controls the 5-Fu loading rates (Figure 5) [39]. By prolonging the time, another stage (Figure 5) has been observed that indicates the presence of different regulating mechanisms, including the influence of the layered loading operations and the 5-Fu diffusion reactions. Finally, the third stage was recognized as the dominant phase during the equilibrium states of BE, EXBE, and Mth/EXBE. This demonstrates that the loaded 5-Fu ions have occupied or consumed all of the functional binding sites (Figure 5) [11,36]. In this stage, several types of processes, including molecular attraction and/or interionic attraction, can direct the loading behaviors [35].

Kinetic Modeling

**Figure 6:**
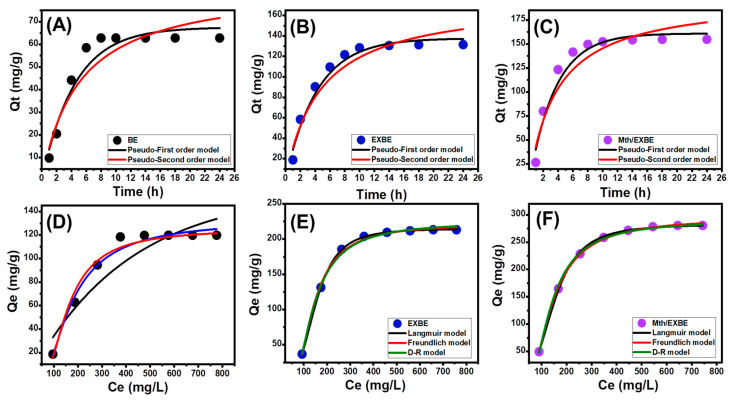
Fitting of the 5-Fu loading processes with the kinetic models (BE (**A**), EXBE (**B**), and Mth/EXBE (**C**)) and classic isotherm models (BE (**D**), EXBE (**E**), and Mth/EXBE (**F**)).

The kinetic properties of the performed 5-Fu loading reactions utilizing BE, EXBE, and Mth/EXBE have been illustrated by utilizing the kinetic assumptions of two independent models, the pseudo-first-order model (P.F.) Equation (4) and the pseudo-second-order (P.S.) model Equation (5). By using nonlinear regression to fit the measured results with both of the models’ illustrative equations, the extent of the match between the load-dependent behaviors and the kinetic hypotheses was assessed (Figure 6A–C; Table 1). The coefficient of correlation (R^2^) and chi-squared (χ^2^) were used as key indicators of the degree of fitting.

(4) $$Q_{t\;}=Q_{e}\;\left(1-e^{-k_{1}\cdot t}\right)$$

(5) $$Q_{t}=\frac{Q_{e}^{2}k_{2}t}{1+Q_{e}k_{2}t}$$

The P.F. model’s kinetic properties more accurately depict the activities of 5-Fu loading into the BE, EXBE, and Mth/EXBE compared to the P.S. model, according to the calculated values of R^2^ and χ^2^. Further support for the aforementioned fitting results appeared from the establishment of an agreement between experimentally determined equilibrium capacities (BE (62.8 mg/g), EXBE (131.5 mg/g), and Mth/EXBE (154.8 mg/g)) and mathematically predicted values of the P.F. model (BE (67.5 mg/g), EXBE (137.6 mg/g), and Mth/EXBE (160.9 mg/g)) (Table 1). According to the kinetic properties of the conventional P.F. model, the loading of 5-Fu into BE, EXBE, and Mth/EXBE was predominantly accomplished by physical processes involving van der Waals forces and/or electrostatic attractions [40,41]. However, the P.F. model describes the loading processes more accurately than the P.S. model, but the fitting results are still in very good agreement with the P.S. model. As a result, it was expected that weak chemical processes like hydrogen binding, electron sharing, hydrophobic bonds, and chemical-based complexes would have a minor impact or support influence during the loading of 5-Fu into BE, EXBE, and Mth/EXBE [36,41]. The combination of the two processes resulted in the development of a chemically loaded layer of medicine, followed by the building of a physically loaded layer using the first one as a base [42].

##### Isotherm Properties

Classic Isotherm Models

Using the Langmuir Equation (5) and Freundlich Equation (6) theories, as well as the Dubinin–Radushkevich (D-R) Equation (7) hypothesis, the equilibrium characteristics of the reactions of 5-Fu loading into BE, EXBE, and Mth/EXBE as potential carriers have been discussed. Nonlinear fitting of the data was performed using the illustrated equations of these models, and the degree of fit was assessed using the correlation coefficient (R^2^) and chi-squared (χ^2^) values (Figure 6D–F; Table 1).

(6) $$Q_{e}=\frac{Q_{max}\;bC_{e}}{\left(1+bC_{e}\right)}$$

(7) $$Q_{e}=K_{f}C_{e}^{1/n}$$

(8) $$Q_{e}=Q_{m}e^{-\beta \varepsilon^{2}}$$

The loading of 5-Fu into EXBE and Mth/EXBE demonstrates the equilibrium properties of the Langmuir isotherm instead of the Freundlich assumption, which is consistent with the determined values of the main model-fitting parameters, in contrast to those of BE that display a greater preference for the Freundlich isotherm properties. As a result, the 5-Fu ions were evenly trapped on the exterior surfaces of EXBE and Mth/EXBE in monolayer layers through multiple broadly and homogeneously dispersed active sites [11,32]. Moreover, RL values that are below 1 indicate that 5-Fu ions preferentially encapsulate into BE, EXBE, and Mth/EXBE carriers. Additionally, the Langmuir isotherm’s numerical parameters were used to compute the theoretical maximum 5-Fu loading capabilities of BE, EXBE, and Mth/EXBE. These values were found to be 183.8 mg/g, 215.1 mg/g, and 282.8 mg/g, respectively.

Regardless of whether the surfaces of BE, EXBE, and Mth/EXBE are homogeneous or heterogeneous, the isothermal characteristics of the examined D-R model may provide insight into the energetic heterogeneity of these potential carriers of 5-Fu. The predominant loading mechanisms, whether chemical or physical in type, are highlighted by the Gaussian energy (E), which has been determined as an obtainable mathematical parameter of the D-R model. The E values of the chemical mechanisms are more than 16 kJ/mol, but those of the physical mechanisms are less than 8 kJ/mol. Gaussian energy levels from 8 to 16 kJ/mol indicate complex processes (physical and chemical) or weak chemical loading processes [11,43]. The 5-Fu loading activities of BE, EXBE, and Mth/EXBE had Gaussian energies of 10.2 kJ/mol, 8.22 kJ/mol, and 7.85 kJ/mol, respectively (Table 1). The findings indicated that, in addition to the hypothesized influence of ion exchange mechanisms (0.6 kJ/mol to 25 kJ/mol), the complex physical and weak chemical process that occurred during the loading of 5-Fu had a major impact. The declination in the values after the exfoliation and methanol functionalization processes signifies the reduction in the impact of the ion exchange process or intercalation between the bentonite layers. Moreover, the decrease in the quantities of the formed chemical complexes and/or hydrogen bonding after the methanol hybridization step directs the reactions in the physical direction by enhancing the electrostatic attractions.

Advanced Isotherm Models

**Figure 7:**
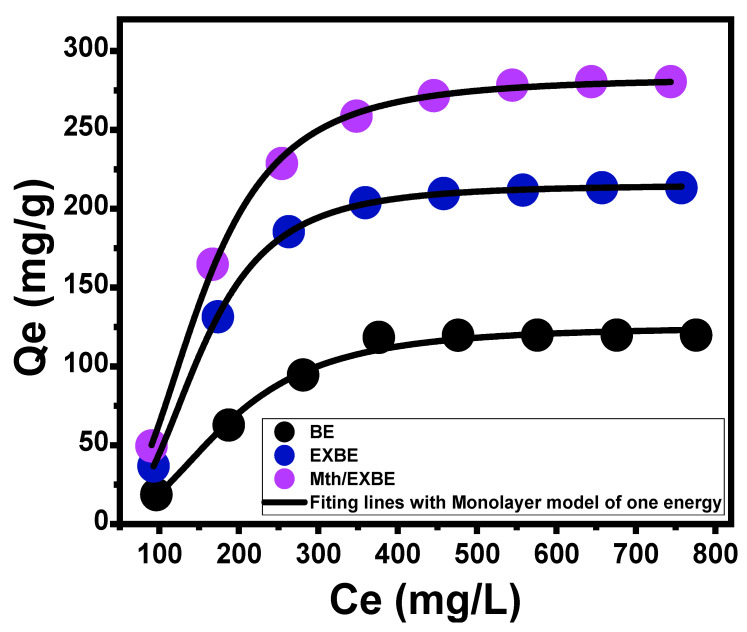
Fitting of the 5-Fu loading processes by BE, EXBE, and Mth/EXBE with advanced monolayer model of one energy site (black lines are the fitting curves with the model).

The authorized advanced equilibrium models, which are based on the isotherm basics of statistical physics theory, provide more insight into BE, EXBE, and Mth/EXBE as 5-Fu carriers in terms of their surface/drug solution interfaces. An advanced monolayer model with one energy Equation (9) and its associated theoretically obtained parameters, either steric or energetic, was used to study the loading characteristics and their controlled mechanistic activities (Figure 7; Table 1). The root mean square error (RMSE) and the determination coefficients (R^2^) have been established to be the main indicators of the fitting degrees.

(9) $$Q=nN_{o}=\frac{nN_{M}}{1+{\left(\frac{C1/2}{Ce}\right)}^{n}}=\frac{Q_{o}}{1+{\left(\frac{C1/2}{Ce}\right)}^{n}}$$

The model’s theoretically calculated steric parameters included the total number of 5-Fu ions that were loaded into the active site (n _(5-Fu_), the density of the fully occupied sites throughout BE, EXBE, and Mth/EXBE (Nm _(5-Fu)_), and their loading capacities at the saturation points (Qsat _(5-Fu)_). The energetic parameter that was assessed was the identified 5-Fu loading energy (E). As a consequence of the modification of EXBE and Mth/EXBE, the computed density of the interaction loading sites (Nm _(5-Fu)_) expanded greatly from 44.9 mg/g (BE) to 66.2 mg/g (EXBE) and 95.9 mg/g (Mth/EXBE). This might be due to the rise in the interaction interfaces following the exfoliation steps, which increases the total number of sites filled with 5-Fu, as well as the integration of extra-active functional groups corresponding to hybridization with methanol. The modification methods greatly increased the predicted 5-Fu loading capacities of BE, EXBE, and Mth/EXBE during their saturation phases, increasing the Qsat from 124.9 mg/g for BE to 214.4 mg/g and 282.6 mg/g for EXBE and Mth/EXBE, respectively. The reckonable quantities of the 5-Fu ions, which were already loaded for each effective site of BE, EXBE, and Mth/EXBE (n _(5-Fu)_), further emphasize the significant impact of modification techniques on the properties of the BE as a medication carrier, particularly after the exfoliation modification step. Theoretically, the number of 5-Fu ions that are loaded for each site of BE, EXBE, and Mth/EXBE (n _(5-Fu)_) is equivalent to 2.78, 3.24, and 2.95, respectively. These values are less than 1, indicating that these ions are vertically loaded on their outer surfaces and entrapped via multi-molecular processes [44,45].

Based on the theoretically anticipated remaining 5-Fu concentrations during the half saturation stages (*C*_1/2_) of BE, EXBE, and Mth/EXBE and the solubility degree of 5-Fu in water, the loading energies (*E*) have been determined using Equation (10) (Table 1).

(10) $$\Delta E=-RT\;ln\left(\frac{S}{C_{1/2}}\right)$$

The 5-Fu loading energies for BE, EXBE, and Mth/EXBE were found to be 8.4 KJ/mol, 9.2 KJ/mol, and 7.86 KJ/mol, respectively. These results verify previously reported findings regarding the processes of the physical loading of 5-Fu into BE, EXBE, and Mth/EXBE (Δ*E* ≤ 40 kJ/mol) [44]. Van der Waals forces (Δ*E* = 4 to 10 kJ/mol), dipole forces (Δ*E* = 2 to 29 kJ/mol), and hydrogen bonding (Δ*E* < 30 kJ/mol) may all be involved in these processes [46,47].

### 3.3. In Vitro Release Profiles

**Figure 8:**
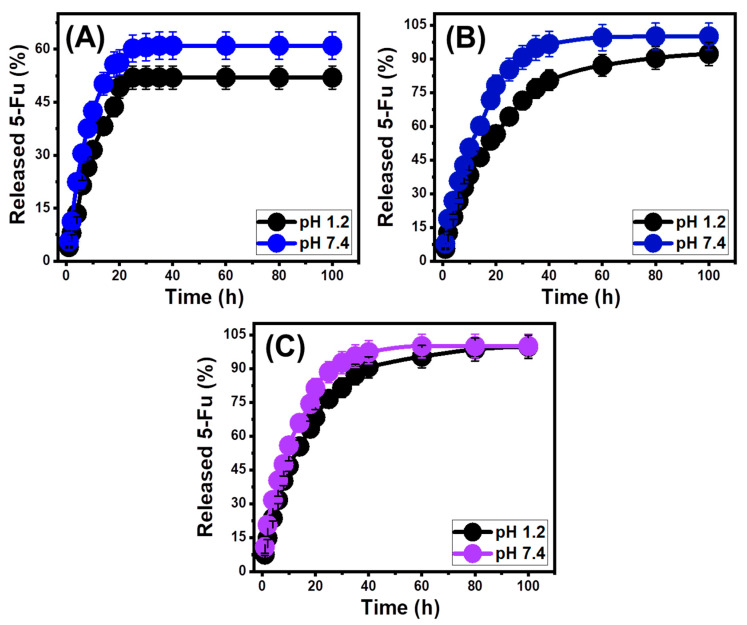
The in vitro release profiles of 5-Fu drug from BE (**A**), EXBE (**B**), and Mth/EXBE (**C**) (the lines are normal plots for the trends of the release results).

The amounts of 5-Fu ions that moved through the gastric fluid (pH 1.2) and intestinal fluid (pH 7.4), which were used to mimic the conditions of cancer cells, were used to study the BE, EXBE, and Mth/EXBE release patterns (Figure 8). The measured percentages of 5-Fu diffusing from BE, EXBE, and Mth/EXBE in the two buffers under study show that the detected rates change with a longer time of release (Figure 8A–C). The rapid 5-Fu releases from BE, EXBE, and Mth/EXBE correlate with considerable variations in the observed 5-Fu released amounts. The observable 5-Fu diffusion rates dramatically decreased after particular release durations, with no apparent increase in the released amounts (Figure 8). The release responses of BE, EXBE, and Mth/EXBE were stabilized at this point. The rapid 5-Fu diffusion that was noticed during early release intervals has been credited to the abrupt desorption of weakly bonded and also physically entrapped 5-Fu ions by the loading sites of BE, EXBE, and Mth/EXBE [48,49,50]. Following complete desorption of these weakly bonded and superficially trapped 5-Fu ions, their release behaviors were regulated by the drug ions, which were strongly bonded or formed chemical complexes with the chemical structures of the carriers, in addition to the intercalated ions between the layers of BE, EXBE, and Mth/EXBE, which had a negative impact on their apparent diffusion speeds (Figure 8) [5,21,51]. In comparison to pH 1.2 (gastric fluid), pH 7.4 (intestinal fluid) is more favorable for the release performances of 5-Fu ions from BE, EXBE, and Mth/EXBE due to its high ionization and solubility behaviors under basic conditions [16,52].

The simulated patterns of 5-Fu release into gastric and intestinal fluids for BE were maintained over 120 h (Figure 8A). Approximately 50% of the 5-Fu load was successfully liberated from the BE framework after 25 h and 14 h, respectively, at pH 1.2 and pH 7.4 (Figure 8A). No complete release states were detected in the gastric (52%) or intestinal (61%) fluids after 120 h (Figure 8A). The expected hydrogen binding of the trapped 5-Fu ions to the structures of bentonite, as well as the intercalated ions between its layered units, might clarify the low-releasing behavior of BE [53]. At pH 1.2 or pH 7.4, the 5-Fu release patterns of EXBE exhibit characteristics that are quicker than those of BE (Figure 8B). A little more than half of the loaded quantity of 5-Fu seeped out of EXBE after 18 and 10 h at pH 1.2 and pH 7.4, respectively (Figure 8B). After 120 h and 80 h, respectively, the maximum release (94.1%) and complete 5-Fu release in the two investigated fluids were observed (Figure 8B). The release properties of 5-Fu ions were also accelerated as a consequence of the hybridization of EXBE with methanol (Mth/EXBE) (Figure 8C). About 50% of the 5-Fu was successfully liberated from the framework of Mth/EXBE after 14 h at pH 1.2 and 8 h at pH 7.4. The complete release of 5-Fu in gastric and intestinal fluids has been observed at 120 and 60 h, respectively (Figure 8C).

The observed acceleration in the releasing speed after the exfoliation of BE into EXBE and after the methanol functionalization step is attributed to (1) the exfoliation process reducing the quantities of the loaded 5-Fu as trapped ions within the layers of raw bentonite, (2) the exfoliation resulting in activation and enhanced the exposures of the active chemical groups which induce the surficial physically loaded ions, (3) the hybridization of EXBE with organic methanol reducing the number of the formed hydrogen bonds directly between the aluminosilicate structure and 5-Fu, and (4) the functionalized methanol molecules providing the surface of EXBE with extensive negative hydroxyl groups that act as active centers for weak electrostatic attractions with the drug ions [5,13]. The slow and controlled diffusion of 5-Fu as an anticancer drug is recommended in specific cases when there is a requirement for more prolonged interactions and contact between the medicine ions and the malignant cells [5,6]. Additionally, abrupt and speedy delivery methods are suggested in specific cases when certain therapeutic dosages must be provided in a brief period of time. As potential 5-Fu carriers, synthetic EXBE and Mth/EXBE may provide excellent delivery systems with controlled loading and release properties.

### 3.4. Release Kinetic Studies

Kinetic analyses of the 5-F release activities from BE, EXBE, and Mth/EXBE were carried out as markers of the appropriately regulated mechanistic processes. The kinetic modeling of the release reactions was performed based on the assumption of zero-order (Z-O), first-order (F-O), Higuchi (H-G), Hixson–Crowell (H-C), and Korsmeyer–Peppas (K-P) based on the linear regression fitting levels with these investigated models [5].

(11) $$W_{t}-W_{0}=K_{0}\cdot t$$

(12) $$\ln\left(W_{\infty }/W_{t}\right)=K_{1}\cdot t$$

(13) $$W_{t}=K_{h}t^{1/2}$$

(14) $$W_{o}^{1/3}-W_{t}^{1/3}=K_{HC}t$$

(15) $$W_{t}/W_{\infty }=K_{p\;}t^{n}$$

According to the zero-order kinetics, the release activities of 5-Fu from BE, EXBE, and Mth/EXBE occur at steady rates, and the loading doses have no discernible impact on the behavior [4]. With regard to F-O kinetics, the 5-Fu dosages loaded into BE, EXBE, and Mth/EXBE show a substantial effect on the efficiency of the release processes [1]. The Higuchi kinetics (H-G) assumes that the diffusion processes have an essential influence on drug release systems [1,54]. The diffusion process, according to the Higuchi hypothesis, was carried out at a steady rate that was slower than the loaded quantity of 5-Fu. Additionally, the used carriers must possess sink properties, and their solubilities and swelling characteristics have an ignored impact on the releasing behaviors [4]. The Hixson–Crowell model’s (H-C) supposition relies on erosion mechanisms instead of diffusion, and the efficiency of the erosion reactions depends significantly on the surface area and grain diameter of BE, EXBE, and Mth/EXBE [4,55]. With regard to the mechanistic hypothesis of Korsmeyer–Peppas kinetics, the release implies a combination of diffusion and erosion processes [1,56].

**Figure 9:**
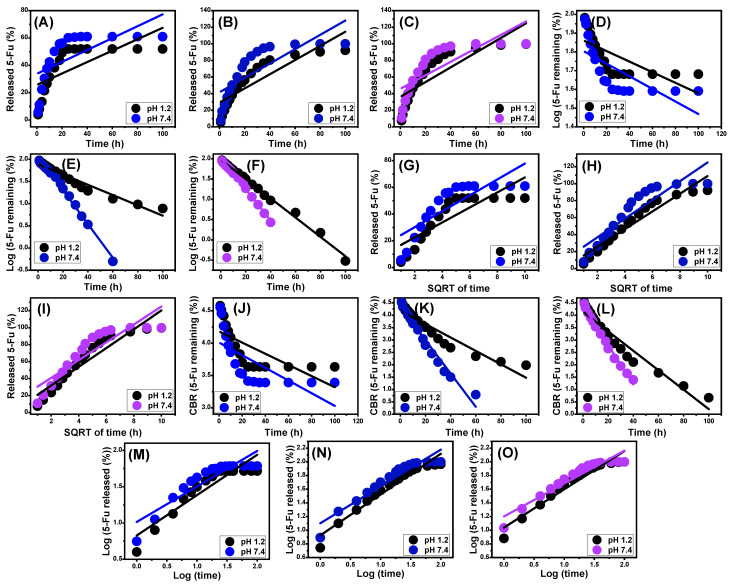
Fitting of the 5-Fu release results with zero-order model (BE (**A**), EXBE (**B**), and Mth/EBE (**C**)), first-order model (BE (**D**), EXBE (**E**), and Mth/EBE (**F**)), Higuchi model ((BE (**G**), EXBE (**H**), and Mth/EXBE (**I**)), Hixson–Crowell model (BE (**J**), EXBE (**K**), and Mth/EXBE (**L**)), and Korsmeyer–Peppas model (BE (**M**), EXBE (**N**), and Mth/EXBE (**O**)) (the linear lines are the regression fitting lines).

According to the coefficients of determination (R^2^), the documented 5-Fu release from BE, EXBE, and Mth/EXBE more closely corresponds to the properties of the F-O kinetics (Figure 9D–F; Table 1) as compared to the Z-O kinetics (Figure 9A–C). This finding suggests that the loaded 5-Fu amounts have a significant influence on the efficacy of their release from their host carriers. Excellent agreement between the release processes and both Higuchi (H-G) (Figure 9G–I; Table 1) and Hixson–Crowell (H-C) (Figure 9J–L; Table 1) models was verified by the determined fitting degrees. These kinetic assessment results demonstrated that the diffusion and erosion mechanisms operated cooperatively during the 5-Fu release periods. The complicated mechanistic theory was corroborated by the reported significant fitting degrees with the Korsmeyer–Peppas model and the derived values of the diffusion exponent (n) (Figure 9M–O; Table 1). The diffusion exponent (n) values exceed 0.45, showing that the BE, EXBE, and Mth/EXBE delivery systems possess non-Fickian transport characteristics [13].

### 3.5. Cytotoxicity Properties

**Figure 10:**
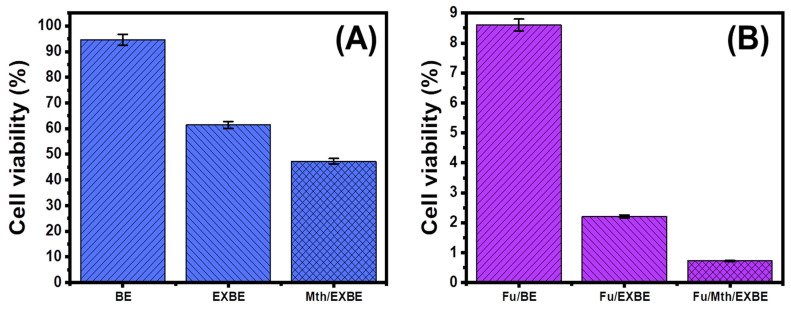
The cytotoxicity effects of BE, EXBE, and Mth/EXBE as free particles (**A**) and their 5-Fu-loaded products (**B**) on colorectal cancer cell (HCT-116) (500 µg/L as suspensions in 50 µL).

HCT-116 cancer cells were used to investigate the cytotoxicity of BE, EXBE, and Mth/EXBE as free particles as well as after their loading with 5-Fu. The free products exhibit considerable cytotoxicity toward the tumor cells, particularly at the highest dosages (500 µg/L), exhibiting cell viability values of 94.6% (BE), 61.4% (EXBE), and 47.3% (Mth/EXBE). As a result, malignant cells are inhibited by 5.4% (BE), 38.6% (EXBE), and 52.7% (Mth/EXBE) (Figure 10). Such significant cytotoxic qualities could be attributed to the excellent surface reactivity of bentonite as single and separated nanolayers, in conjunction with the oxidation properties of the clay nanostructures due to their metal and metal oxide impurities. The 5-Fu-loaded BE, EXBE, and Mth/EXBE had enhanced cytotoxicity and anticancer activity when tested against the human HCT-116 malignant cell line. The cell viability, IC-50, and inhibitory percentage of 5-Fu-loaded BE (500 µg/mL) were determined to be 8.6%, 101.7 µg/L, and 91.4%, respectively (Figure 10). The synthesized EXBE’s anticancer properties have been greatly enhanced, as evidenced by the observed results (2.21% (cell viability), 6.14 µg/mL (IC-50), and 97.79% (inhibitory percentage)) (Figure 10). Similarly, the results obtained for the 5-Fu-loaded Mth/EXBE were markedly enhanced to 0.73% (cell viability), 99.27% (inhibitory percentage), and 3.0 µg/mL (IC-50) (Figure 10). These findings reveal significant enhancements in cellular toxicity and anticancer activity after the loading of 5-Fu into the studied carriers, especially the modified forms of bentonite (EXBE and Mth/EXBE), in addition to the previously recognized controlling effects on loading and release behaviors.

## 4. Conclusions

Natural bentonite clay was successfully exfoliated into individual sheets (EXBE) and hybridized by organic methanol molecules (Mth/EXBE) as a potential carrier of 5-fluorouracil with enhanced loading and release properties. The exfoliation and hybridization steps resulted in insignificant enhancements in the surface area and the numbers of effective loading sites to 66.2 mg/g and 95.9 mg/g, respectively, as compared to 44.9 mg/g for BE. This strongly improved the loading capacities from 124.9 mg/g (BE) to 214.4 mg/g (EXBE) and 282.6 mg/g (Mth/EXBE). The loading occurred due to multi-molecular and complex physical and weak chemical mechanisms, considering both the estimated Gaussian and 5-Fu loading energies. The 5-Fu release patterns of EXBE and Mth/EXBE continued at slow rates for about 100 h and are characterized by non-Fickian transport characteristics that indicate the operation of both diffusion and erosion release mechanisms. Concerning their cytotoxic impacts on the HCT-116 cancer cell line, the 5-Fu-loaded samples had cell viability percentages of 8.6% (ZA), 2.21% (CS/ZA), and 0.73% (CD/ZA).

## Figures and Tables

**Table 1:** The obtained mathematical parameters of the studied kinetic, classic isotherm, advanced isotherm, thermodynamic, and release kinetic models.

Model	Parameters		BE		EXBE		Mth/EXBE
Kinetic models							
Pseudo-first-order	K_1_ (min^−1^)		0.223 ± 0.035		0.233 ± 0.031		0.285 ± 0.042
	Qe _(Cal)_ (mg/g)		67.5 ± 4.2		137.6 ± 7.19		160.9 ± 8.3
	R^2^		0.95		0.97		0.96
	X^2^		0.73		1.08		1.53
Pseudo-second-order	k_2_ (g mg^−1^ min^−1^)		0.0022 ± 0.0001		0.0011 ± 0.0004		0.0013 ± 0.0005
	Qe _(Cal)_ (mg/g)		86.87 ± 6.8		175.7 ± 10.8		198.9 ± 12.87
	R^2^		0.92		0.94		0.92
	X^2^		1.35		2.04		2.92
Isotherm models							
Langmuir	Q_max (mg/g)_		183.8 ± 4.65		215.1 ± 8.13		282.8 ± 10.52
	b (L/mg)		2.8 × 10^−3^ ± 0.0011		8.35 × 10^−8^ ± 1.23 × 10^−8^		3.64 × 10^−7^ ± 1.11 × 10^−8^
	R^2^		0.76		0.98		0.99
	X^2^		5.47		0.12		0.076
Freundlich	1/n		0.702 ± 0.042		0.43 ± 0.051		0.48 ± 0.027
	k_F_ (mg/g)		1.215 ± 0.23		4.7 ± 1.16		5.34 ± 1.07
	R^2^		0.89		0.98		0.99
	X^2^		2.31		0.18		0.092
D-R model	β (mol^2^/kJ^2^)		0.0042 ± 0.0011		0.0074 ± 0.0017		0.0081 ± 0.0022
	Q_m_ (mg/g)		125.5 ± 2.73		224.2 ± 4.49		289.7 ± 6.16
	R^2^		0.98		0.99		0.99
	X^2^		0.49		0.09		0.06
	E (kJ/mol)		10.2 ± 2.87		8.22 ± 1.64		7.85 ± 1.33
Monolayer model of one energy	n		2.78 ± 0.196		3.24 ± 0.0195		2.95 ± 0.064
	Nm (mg/g)		44.96 ± 3.97		66.2 ± 0.465		95.9 ± 2.51
	Q_(sat)_ (mg/g)		124.9 ± 6.74		214.4 ± 6.39		282.6 ± 8.39
	∆E (kJ/mol)		8.4 ± 1.82		9.2 ± 1.88		7.86 ± 1.17
Release kinetics							
Models			Determination coefficient				
	BE		EXBE			Mth/EXBE	
	Acetate buffer (pH 1.2)	Phosphate buffer (pH 7.4)	Acetate buffer (pH 1.2)	Phosphate buffer (pH 7.4)		Acetate buffer (pH 1.2)	Phosphate buffer (pH 7.4)
Zero-order	0.39	0.36	0.73	0.59		0.67	0.56
First-order	0.42	0.41	0.94	0.99		0.99	0.99
Higuchi	0.60	0.56	0.91	0.83		0.88	0.81
Hixson–Crowell	0.40	0.38	0.92	0.96		0.93	0.99
Korsmeyer–Peppas	0.75	0.73	0.94	0.90		0.93	0.90
n	0.62	0.55	0.59	0.54		0.55	0.48

## Data Availability

Data are available from the corresponding authors upon reasonable request.
